# Evolution of COVID-19 in patients with autoimmune rheumatic diseases

**DOI:** 10.18632/aging.202193

**Published:** 2020-12-03

**Authors:** Rongrong Pang, Jun Zhao, Zhenhua Gan, Zhiliang Hu, Xiang Xue, Yanjun Wu, Qinghua Qiao, Aifang Zhong, Xinyi Xia, Hui Liao, Zhihua Wang, Libo Zhang

**Affiliations:** 1 COVID-19 Research Center, Institute of Laboratory Medicine, Jinling Hospital, Nanjing University School of Medicine, The First School of Clinical Medicine, Southern Medical University, Nanjing 210002, Jiangsu, China; 2 Department of Laboratory Medicine, Nanjing Red Cross Blood Center, Nanjing 210003, Jiangsu, China; 3 Graduate School, Nanjing Medical University, Nanjing 211166, Jiangsu, China; 4 Department of Medical Administration, Jinling Hospital, Nanjing University School of Medicine, Nanjing 210002, China; 5 Joint Expert Group, Wuhan Huoshenshan Hospital, Wuhan 430100, Hubei, China; 6 Nanjing Infectious Disease Center, The Second Hospital of Nanjing, Nanjing University of Chinese Medicine, Nanjing 210003, Jiangsu, China; 7 School of Public Health, Nanjing Medical University, Nanjing 211166, Jiangsu, China; 8 Department of Biochemistry and Molecular Biology, University of New Mexico, Albuquerque, NM 87131, USA; 9 Department of Information, Jinling Hospital, Nanjing University School of Medicine, Nanjing 210002, China; 10 Department of Information, Wuhan Huoshenshan Hospital, Wuhan 430100, Hubei, China; 11 Medical and Technical Support Department, Pingdingshan Medical District, The 989th Hospital, Pingdingshan 467000, Henan, China; 12 Department of Laboratory Medicine and Blood Transfusion, Wuhan Huoshenshan Hospital, Wuhan 430100, Hubei, China; 13 Medical Technical Support Division, Changzhou Medical District, The 904th Hospital, Changzhou 213003, Jiangsu, China; 14 Department of Hematology, The Air Force Hospital from Eastern Theater of PLA, Nanjing 210002, Jiangsu, China; 15 Department of Laboratory Medicine and Blood Transfusion, The 907th Hospital, Nanping 350702, Fujian, China

**Keywords:** coronavirus disease 2019, COVID-19, SARS-CoV-2 virus, autoimmune rheumatic diseases, systematic lupus erythematosus, rheumatoid arthritis

## Abstract

The characteristics of COVID-19 patients with autoimmune rheumatic diseases (AIRD) have rarely been reported. Patients with AIRD have suppressed immune defense function, which may increase their susceptibility to COVID-19. However, the immunosuppressive agents AIRD patients routinely used may be beneficial for protecting the cytokine storm caused by SARS-CoV-2. In this retrospective study, we included all confirmed cases in Huoshenshan Hospital from February 4 to April 9. Data were extracted from electronic medical records and were analyzed for clinical and laboratory features using SPSS (version 25.0). Of 3059 patients, 21 had the comorbidities with systematic lupus erythematosus (SLE) and/or rheumatoid arthritis (RA), including 5 with SLE, 15 with RA, and 1 with Rhupus. The proportion was 57.1% for severe cases, 61.9% for either severe or critical cases, and 4.8% for critical cases. The main manifestations, ARDS and ICU admission rate, as well as the mortality and length of hospital stay of COVID-19 in AIRD patients were similar to COVID-19 patients in the general population. Our preliminary experience shows that patients with AIRD tend to have a higher risk of SARS-CoV-2 infection, and may be at risk for a severe but less likely critical disease course. Further investigation is needed to understand the immunological features of these diseases.

## INTRODUCTION

Currently we are on a global pandemic of coronavirus disease-2019 (COVID-19). Up till today, no pharmacological therapies have been proven efficacy yet. The main treatments are supportive therapies, including fluid management, oxygen supply and extracorporeal membrane oxygenation. Accordingly, the resolution and recovery rely heavily on the host's immunity.

However, the host immunity acts like a double-edge sword during the response to the new coronavirus: it may provide a strong immunological response adequate for virus clearance, while on the other hand, excessive proinflammatory cytokines induced by the uncontrolled immune response with features of cytokine storm may cause a severe disease course and then worsen the prognosis of COVID-19 [1–4].

People with autoimmune rheumatic diseases (AIRD), such as systematic lupus erythematosus (SLE) and rheumatoid arthritis (RA), were inferred to have an increased risk of SARS-CoV-2 infection due to suppressed immune defense function by the illness per se or the therapy, since precedent studies reported that immune suppression delayed viral clearance and increased rates of secondary infections in SARS and MERS outbreaks [5, 6].

Nonetheless, it remains controversial that whether individuals with immunosuppression are more susceptible of or protective from SARS-CoV-2 infection. A previous study showed lack of symptomatic infection with SARS-CoV-2 in children treated with immunomodulatory and immunosuppressive treatment [7]. Similar findings in a cohort of liver transplant patients reported a low rate (3 out of 200) of SARS-CoV-2 infections [8]. However, to date, little evidence-based data were available to describe the clinical and immunological features of AIRD in COVID-19 patients.

As a result, concerns have arisen about the characteristics, diagnosis, treatment and monitoring of certain diseases. Here we describe the clinical characteristics, laboratory findings and outcome of 21 COVID-19 patients underlying comorbidities with AIRD from Huoshenshan Hospital.

## RESULTS

### Baseline characteristics and laboratory findings

The underlying autoimmune diseases in this study contained 5 cases of SLE, 15 cases of RA and 1 case of Rhupus. Twenty (95.2%) patients were female. The median age was 62 years old (50.5-66.5). Totally, fourteen (66.7%) patients had other comorbidities (Table 1). Five patients had more than two kinds of comorbidities, in which 4(80%) cases were either severe or critical illness. While in the 16 cases who had no more than one comorbidity, only 6 (37.5%) patients developed severe illness.

**Table 1:** Comorbidities of patients with AIRD infected with SARS-CoV-2^f^.

**Characteristics**	**Types of AIRD**			**Total**
	**SLE (n=5)**	**RA (n=15)**	**Rhupus (n=1)**	**(n=21)**
Female, n (%)	4(80)	15(100)	1(100)	20(95.2)
Age, years, median (IQR)	48(35-52)	64(58-68)	66	62(50.5-66.5)
**Comorbidities**				
Pre-existing pulmonary diseases^a^, n (%)	0(0)	0(0)	1(100)	1(4.8)
Cardiovascular and cerebrovascular diseases^b^, n (%)	4(80)	3(20)	0(0)	7(33.3)
Diabetes, n (%)	0(0)	2(13.3)	1(100)	3(14.3)
Kidney diseases^c^, n (%)	2(40)	1(6.7)	1(100)	4(19.0)
Hypothyroidism, n (%)	2(40)	0(0)	0(0)	2(9.5)
Tumors^d^, n (%)	1(20)	0(0)	0(0)	1(4.8)
Other comorbidities^e^, n (%)	3(60)	4(26.6)	1(100)	8(38.1)

^a^The only Rhupus patient had a pre-existing pulmonary disease of bronchiectasis with infection.

^b^Cardiovascular and cerebrovascular diseases included primary or secondary hypertension, coronary atherosclerosis, lacunar infarction, uremic cardiomyopathy, post congenital heart surgery, and pericardial effusion.

^c^Kidney diseases included primary or secondary nephritis and chronic kidney disease.

^d^One patient with SLE had an adrenal tumor.

^e^Other comorbidities included primary or secondary anemia, hypoproteinemia, hyponatremia, gout or hyperuricemia, thrombocytopenia, disorders of electrolyte metabolism, pleural or abdominal effusion, chronic pharyngitis, stomach ulcer, gallstone and multiple organ failure.

^f^Data were expressed as number (percentage) and median (interquartile range).

The median time from symptoms onset to admission was 25 (14-30) days. At the time of admission, 90.5% (19/21) presented with fever, with the highest temperature of 39.8° C. 71.4% (15/21) had cough; 38.1%(8/21) had chills; 19.0% (4/21) had sputum; 42.8% (9/21) had shortness of breath; 66.7% (14/21) had myalgia; 14.3% (3/21) patients had chest pain, and nausea was found in 61.9%(13/21) patients.

Laboratory results showed that 60% of the patients with SLE, 33.3% with RA and the only one Rhupus patient developed lymphopenia, while 28.6% (6/21) had lymphopenia at entry. The median level of lymphocyte counts at entry was 1.41×10^9^/L. C-reactive protein was significantly increased in 80% SLE patients and 40% RA patients, as well as the Rhupus patient. In addition, elevated PCT and IL-6 were also demonstrated among the patients in a certain proportion (Table 2).

**Table 2:** Clinical characteristics, treatment, outcome and laboratory findings of patients with AIRD infected with SARS-CoV-2^a^.

**Characteristics**	**Types of AIRD**			**Total**
	**SLE (n=5)**	**RA (n=15)**	**Rhupus (n=1)**	**(n=21)**
**Clinical characteristics**				
Temperature on admission, ° C median (IQR)	39.0(38.4-39.2)	38.0(37.8-39.0)	38.2	37.9(38.5-39.0)
Respiratory rate, median (IQR)	22(20-22.5)	20(19-21)	20	20(20-22)
Pulse, median (IQR)	101(91-106.5)	84(76-100)	80	92(76-101.5)
Fever, n(%)	5(100)	13(86.6)	1(100)	19(90.5)
Cough, n(%)	3(60)	11(73.3)	1(100)	15(71.4)
Chills, n(%)	2(40)	6(40)	0(0)	8(38.1)
Sputum, n(%)	1(20)	2(33.3)	1(100)	7(33.3)
Shortness of breath, n(%)	2(40)	6(40)	1(100)	9(42.9)
Myalgia, n(%)	2(40)	11(73.3)	1(100)	14(66.7)
Chest pain, n(%)	1(20)	1(6.6)	1(100)	3(14.3)
Nausea, n(%)	2(40)	10(66.6)	1(100)	13(61.9)
**Treatment of COVID-19**				
Oxygen support, n(%)	2(40)	4(80)	1(100)	7(33.3)
Invasive mechanical ventilation, n(%)	0(0)	0(0)	0(0)	0(0)
Interferon, n(%)	1(20)	2(33.3)	0(0)	3(14.3)
Arbidol Hydrochloride Capsules, n(%)	2(40)	10(66.6)	1(100)	13(61.9)
Oseltamivir, n(%)	0(0)	2(33.3)	0(0)	2(9.5)
Thymalfasin, n(%)	0(0)	3(20)	0(0)	3(14.3)
Lianhuaqingwen capsule, n(%)	1(20)	10(66.6)	0(0)	11(52.4)
Other Chinese herbal medicines, n(%)	1(20)	1(6.6)	0(0)	2(9.5)
IVIG treatment, n(%)	1(20)	0(0)	0(0)	1(4.8)
CPT treatment, n(%)	2(40)	0(0)	0(0)	2(9.5)
Antibiotic treatment, n(%)	3(60)	8(53.3)	1(100)	12(57.1)
**Treatment of autoimmune diseases**	
Corticosteroid treatment during hospitalization, n(%)	2(40)	3(20)	1(100)	6(28.6)
Long-term Corticosteroid treatment, n(%)	5(100)	1(6.6)	1(100)	7(33.3)
Hydroxychloroquine treatment, n(%)	4(80)	1(6.6)	1(100)	6(28.6)
Cyclosporin A, n(%)	1(20)	0(0)	0(0)	1(4.8)
Mycophenolate Mofetil, n(%)	1(20)	0(0)	0(0)	1(4.8)
**Outcome of COVID-19**	
Discharged, n(%)	4(80)	15(100)	1(100)	20(95.2)
Hospital stay, days, median (IQR)^#^	10(8-17)	13(8-23)	16	13(8.5-19.5)
Onset to discharge, days, median (IQR)^#^	22(15.5-57)	42(36-53)	33	42(28-52)
Died, n(%)	1(20)	0(0)	0(0)	1(4.8)
**Laboratory findings**	
Lymphocyte counts at entry,×10^9^/L, median (IQR)	1.23(0.62-1.46)	1.49(1.09-1.58)	1.77	1.41(1.04-1.58)
Lymphopenia, n(%)	3(60)	5(33.3)	1(100)	9(42.8)
High CRP, n(%)	4(80)	6(40)	1(100)	11(52.4)
High PCT, n(%)	3(60)	3(20)	1(100)	7(33.3)
High IL-6, n(%)	3(60)	4(26.6)	0(0)	7(33.3)
Low C3, n(%)	3(20)	0(0)	0(0)	3(14.3)

^#^Excluding the deceased patient.

^a^Data were expressed as number (percentage) and median (interquartile range).

### Treatment

The main treatments included supportive therapy, antibiotic and antiviral therapy, as well as immunotherapy. The antiviral regimen mainly included interferon-alpha (5 million U atomized twice daily), arbidol (200mg three times a day orally, for no more than 10 days), lopinavir/ritonavir (400mg/100mg twice daily, for no more than 10 days), and the traditional Chinese medicine such as Lianhuaqingwen capsule was also employed depends on the actual situation of the patient. Convalescent plasma therapy was suitable for severe and critical patients with rapid progression. Patients with SLE and Rhupus remained on immunomodulatory or immunosuppressive therapy in order to control their underlying rheumatic conditions. Corticosteroids were administrated to 6 patients during their hospitalization, one patient received intravenous immunoglobulin treatment, and three patients recovered under the therapy of convalescent plasma transfusion.

During their hospitalization, two patients of SLE remained in stable condition. One patient of SLE had hydrothorax and pericardial effusion revealed by CT. Thus she was treated with a decreased dose of prednisone acetate tablets (30mg to 20mg). Another patient was treated with an increased dose of methylprednisolone (from 40mg to 60mg per day, intravenous) due to a relapse of SLE, but withhold unchanged dose of immunosuppressant therapy. One patient with RA was given prednisone acetate tablets (20mg, once daily, orally) for her elevated CRP and an underlying inflammatory process revealed by radiology, while another RA patient was administrated with prednisone tablets at a dose of 10mg orally because of a worsened pain of her lower extremity joint. However, none of the patients presented definite worsening or exacerbation of the pre-existed autoimmune diseases secondary to COVID-19.

### Severity and outcome of patients with AIRD infected with SARS-CoV-2

Table 3 shows the severity and outcome markers of the study subjects. Approximately 57.1% (12/21) AIRD subjects were classified as severe cases, while 61.9% (13/21) as either severe or critical cases. In terms of critical cases, the proportion was 4.8% (1/21) in the AIRD population.

**Table 3:** Severity and outcomes of patients with AIRD^a^

	**Subjects with AIRD (n=21)**
Severe cases, n(%)	12(57.1%)
Severe or critical cases, n(%)	13 (61.9%)
Critical cases, n(%)	1 (4.8%)
Length of hospital stay, days, median (IQR)	13.5(7-37)
Developing ARDS, n(%)	1(4.8%)
Admission to ICU, n(%)	0 (0%)
Mortality, n(%)	1(4.8%)

^a^Data were expressed as number (percentage) and median (interquartile range).

Twenty patients have recovered and been discharged from hospital with a median hospital stay of 13.5 (8.25 - 20.25) days and median illness course of 42 (33.75 - 52.5) days. Only one patient developed acute respiratory distress syndrome (ARDS), and unfortunately the patients eventually died. He was a 25-year-old man with obesity and complex underlying comorbidities including lupus nephritis, chronic kidney disease, renal hypertension, renal anemia, uremic cardiomyopathy, and post congenital heart surgery, and he had long term self-medication with immunosuppressant such as prednisone and leflunomide for treating SLE. He subsequently died with multiorgan failure, and cardiopulmonary arrest on day 9 of hospitalization, despite aggressive supportive care and investigational therapies.

## DISCUSSION

We report the clinical characteristics, laboratory findings and outcomes of 21 COVID-19 patients who have comorbidities with SLE and/or RA. Fever, cough, and nausea were observed in most of the patients, which was similar to the manifestation of COVID-19 in the general population [9, 10]. Notwithstanding, it provides us a picture of the characteristics and course of these patients.

A previous study has reported that immunosuppressed subjects may have an increased risk of SARS-CoV-2 infection [11]. In our hospital, five (163 per 100,000) and fifteen (490 per 100,000) patients had a history of SLE and RA, respectively, meanwhile one patient (32.69 per 100,000) had both the symptoms of SLE and RA. The prevalence was remarkably higher than that in the general population(27-70 per 100,000 for SLE, 280 per 100,000 for RA, respectively) [12–15], indicating that AIRD patients might have an increased risk of SARS-CoV-2 infection.

Comorbidities such as hypertension and diabetes are recognized as important factors in patients with COVID-19. Our study demonstrated that patients who had multiple comorbidities were seen to have a higher severity of COVID-19. Thus more varieties of comorbidities were also associated with increased severity in the AIRD group of patients, similar to the general population [16, 17].

In AIRD related patients, the proportion of severe cases(57.1%) was relatively higher than the general population(11.4-15.74%) [18, 19], as well as in either severe or critical cases(61.9% vs. 19%), whereas it was lower in critical cases(4.9% vs. 5%). We proposed that in the early phase, the patients may be prone to more severe infection due to their impaired immune function, which was not adequate for virus clearance. While in the late phase, it is plausible that some immunosuppressive agents routinely used for rheumatological therapy might offer an advantage on the aberrant inflammatory and cytokine response perpetuated by the host immune system, preventing the patients from developing critical illness.

However, our study had several limitations. Firstly, we had a small sample size, which might reflect a local experience. Secondly, outcomes were likely to be mixed depending on factors such as different types and disease activity of AIRD, and also combinations of therapies [7, 20–22]. AIRD patients with COVID-19 are a great challenge for the physicians to achieve personalized treatment programs both effective for COVID-19 and their underlying rheumatic conditions. A striking interplay between infection, inflammation, and immunological pathogenetic mechanisms in COVID-19 patients with AIRD needs to be further studied. Thirdly, the role of anti-rheumatic medications is an ongoing area of interest [23–25]. Drugs including hydroxychloroquine (HCQ), chloroquine (CQ) and glucocorticoids theoretically have potential associations with the severity of COVID-19. However, due to limitations imposed by the small sample size, our data are not sufficient to draw the conclusion whether current treatment for AIRD is a risk or not for severe forms of evolution. There is a need for well-conducted and large population-based studies to ascertain the role of specific rheumatic treatments in the evolution of COVID-19.

In the present work, we retrospectively analyzed the clinical features, laboratory findings and outcomes of 21 COVID-19 confirmed patients accompanying AIRD, and our preliminary experience shows that patients with AIRD tend to have a higher risk of SARS-CoV-2 infection, and may be at risk for a severe but less likely critical disease course.

## MATERIALS AND METHODS

### Study design

This study included 21 COVID-19 patients with underlying autoimmune diseases, who were admitted to the Huoshenshan Hospital, Wuhan, China, from February 4, 2020, to April 9, 2020. The demographic data, medical history, underlying diseases, symptoms, signs, blood laboratory parameters, treatments and outcomes were collected from the electronic health record system and were retrospectively analyzed. The comorbidities evaluated in the study included pre-existing pulmonary diseases, cardiovascular and cerebrovascular diseases, diabetes, kidney diseases, thyroid diseases and tumors. This study was approved by the Medical Ethical Committee of Huoshenshan Hospital with participants’ written informed consent (HSSLL011).

### Severity of COVID-19

Severe disease was defined by one or more of the following conditions: (1) Shortness of breath, RR ≥ 30 times/min; (2) In the resting state, the oxygen saturation is less than or equal to 93%; (3) Arterial oxygen partial pressure(PaO_2_) /oxygen inhalation concentration(FiO_2_) ≤ 300mmHg; (4)Pulmonary imaging shows that patients with obvious lesion progression > 50% within 24-48 hours.

The critical disease was defined by one or more of the following conditions: (1)Respiratory failure occurs, and mechanical ventilation is required; (2)Shock; (3)ICU monitoring and treatment are required for other organ failures.

### Statistical analysis

Continuous variables were summarized as median with interquartile range; categorical variables were summarized as counts and percentages. Data were extracted from electronic medical records and were analyzed for clinical and laboratory features using SPSS (version 25.0).

### Disclosure statement

There are no potential conflicts of interest relevant to this article to report.
